# Cerebral Small Vessel Disease: A Bibliometric Analysis

**DOI:** 10.1007/s12031-022-02070-2

**Published:** 2022-10-06

**Authors:** Wei Ma, Yi-Bao Yang, Ting-Ting Xie, Yi Xu, Na Liu, Xue-Ni Mo

**Affiliations:** 1 Guangxi University of Chinese Medicine, Nanning, China; 2 Ruikang Hospital Affiliated of Guangxi University of Chinese Medicine, Nanning, China; 3 The First Affiliated Hospital of Guangxi, University of Traditional Chinese Medicine, Nanning, China; 4 Maternal and Child Health Care Of Guangxi Zhuang Autonomous Region, Nanning, China

**Keywords:** Cerebral small vessel disease, CiteSpace, Bibliometrics

## Abstract

Cerebral small vessel disease is a common neurological disease, and its incidence is increasing year by year worldwide. In recent years, research on cerebral small vessel disease has gained more and more attention. Our research aims to visualize publications to identify the hotspots and frontiers of cerebral small vessel disease research, and to provide reference and guidance for further research. Publications related to cerebral small vessel disease were searched from the Web of Science Core Collection and screened according to inclusion criteria. CiteSpace 5.8.R3 was used to evaluate and visualize results, including generating web maps and analyzing annual publications, countries, institutions, bibliographic and co-cited references, and keywords; in this article, we use CiteSpace and VOSviewer for the 2012 Cerebral small vessel disease and bibliometric analysis from January 1, 2022 to April 30, 2022. A total of 3037 papers related to cerebral small vessel disease were retrieved, and the number of published papers showed a steady upward trend. Among them, Neuroimaging standards for research into small vessel disease and its contribution to ageing and neurodegeneration, the most symbolic references in the field of cerebral small vessel disease have been cited a total of 438 times. Stroke is the most active journal (227 articles) and USA publishes up to 800 articles. Harvard Med SchUniv Edinburgh (133 papers) and Charidimou (85 papers) are the institutions and authors who have made the most contributions in this field, respectively. Among the keywords, most of them are related to the pathogenesis of cerebral small vessel disease. After 2018, gut-brain axis and cortex are the keywords with the strongest number of cited outbreaks. There is increasing evidence that cerebral small vessel disease is a research frontier and may remain a research hotspot in the future.

## Introduction

Cerebral small vessel disease (CSVD) is a common neurological disease, a group of diseases with small perforating arteries and arterioles (diameter 40–200 μm), capillaries, and venules of the brain as the main lesions. It is the most common cause of vascular dementia, accounting for 25% of ischemic strokes and most hemorrhagic strokes (Agirman et al. 2021). It is commonly associated with Alzheimer’s disease and exacerbates the resulting cognitive impairments, which are responsible for approximately 50% of dementias worldwide (Arba et al. 2019; Bai et al. 2022). In China, lacunar infarction caused by cerebral microvascular disease accounts for 25 to 50% of ischemic stroke, which is higher than that in Western countries (Bordes et al. 2022). It is foreseeable that CSVD will be a major disease affecting the quality of life of the elderly in the future (Thrippleton et al. 2019). Therefore, CSVD will be a great challenge in the field of encephalopathy, and its underlying pathogenesis needs to be further studied.

In recent years, cerebral small vessel disease has gained more attention in various research fields, and numerous academic journals have published articles related to CVSD research. However, few studies have systematically investigated the scientific achievements and current state of the field from a worldwide perspective (Du et al. 2022). Therefore, it is extremely important to use visual analysis to reveal the global status, future research trends, and hotspots of CSVD research. Bibliometric science not only provides quantitative and statistical analysis of publications in a specific field but also accurately reveals the most representative studies. In addition, by presenting massive data in the form of knowledge graphs, researchers can comprehensively analyze the development of a discipline and intuitively understand cutting-edge trends. Through CiteSpace, our surveys are based on the following: national networks, authors, institutions, annual publications, categories and references, and co-occurrence keyword analysis and clustering. Findings can benefit researchers by shaping future research directions and informing policy formulation (Ma et al. 2021; Sabe et al. 2022). Bibliometrics, first defined by Pritchard in 1969, is a visualization method that quantitatively evaluates the contributions of research fields using methods such as mathematics and statistics (Ma et al. 2021). Bibliometrics can not only accurately reveal research trends in a certain field but also an important method to predict research hotspots and frontiers (Sabe et al. 2022). Therefore, we used CiteSpace and VOSviewer to conduct the bibliometric analysis of the research on small vessel disease from 2012 to 2022 to investigate the research progress and hotspots in the field of cerebral small vessel disease and to help researchers quickly understand the research of cerebral small vessel disease The status quo and the development of research strategies to provide some literature data support for researchers’ research on cerebral small vessel disease (Shen et al. 2022).

## Methods

### Source of Bibliometric Data and Search Strategies

**Fig. 1:**
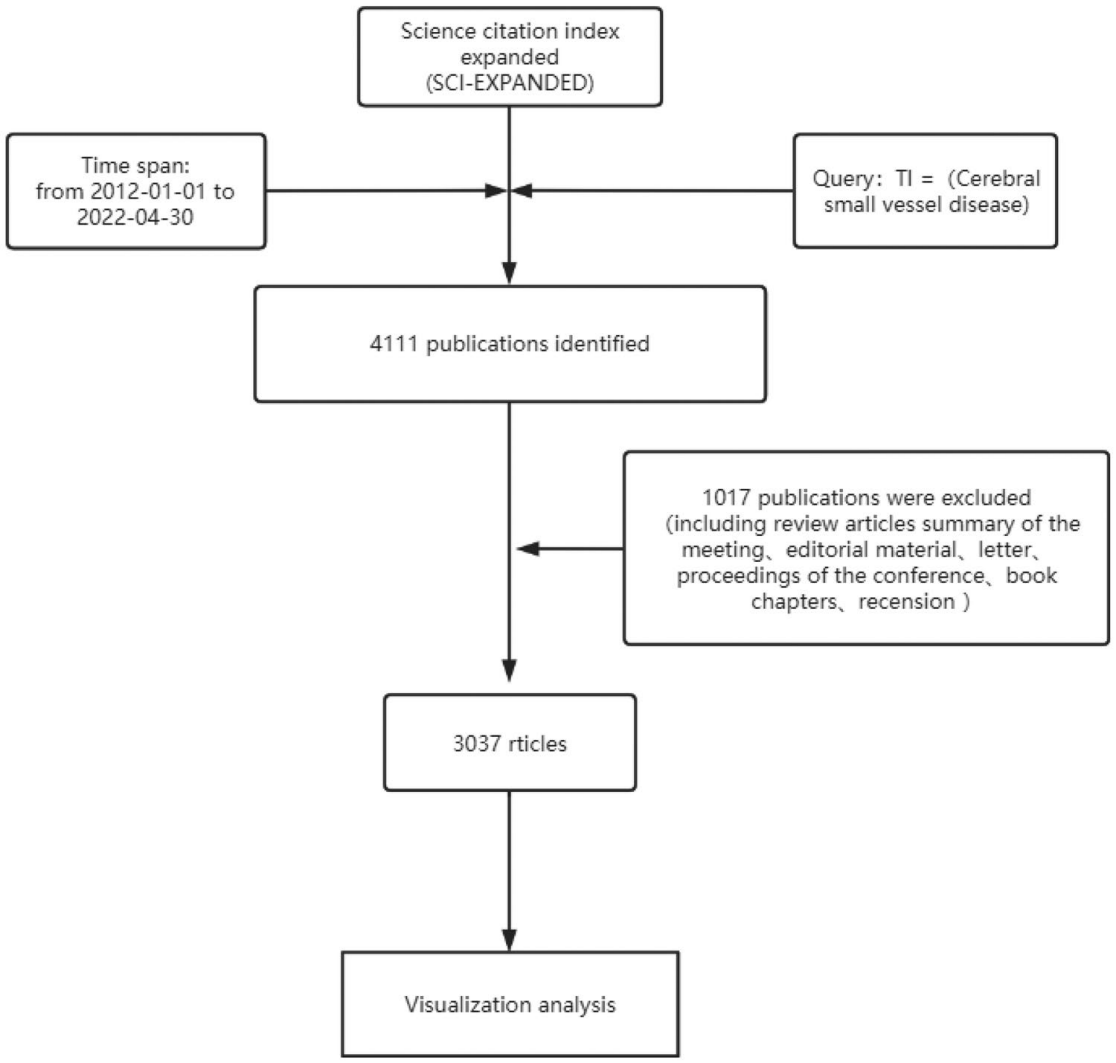
Flow chart of research and analysis of cerebral small vessel disease

Selecting the Web of Science Core Collection (WOSCC) as the main data source for data retrieval, WOSCC is a canonical online database which is considered to be the most suitable database for bibliometric analysis (Yao et al. 2020). The data retrieval time was from January 1, 2012 to April 30, 2022, and the set retrieval formula is [subject = (cerebral small vessel disease)] and [language = (English)] and [year range = (2012–2022)], the search results were de duplicated, and the inclusion criteria were the following: according to the document type, only articles were included, and the language was limited to English. Exclusion criteria were the following: delete conference calls, comments, news, reviews, errata, etc., and delete studies not related to cerebral small vessel disease (Guo et al. 2021). With reference to other studies, the title and abstract were reviewed by two independent researchers. As of April 30, 2022, 3037 publications were finally screened in the Web of Science database. Since the set time span does not include the literature for the 8 months after 2022, the data for 2022 is not complete, and the research analysis does not represent the whole year. Considering that the database is updated regularly, all data are obtained within 1 day to avoid potential discrepancies. The flow chart of the research data analysis is shown in Fig. 1. The data used in this study were downloaded directly from public databases without further animal experiments, so the approval of the ethics committee or informed consent was not required.

### Data Export and Extraction

The WoSCC database is considered the most suitable one for bibliometric analysis and is widely used for biometric analysis and visualization of scientific literature. The search parameters are set as follows: time slice (years per slice) is January 1, 2012 to April 30, 2022, years per slice (1), links (strength = cosine; scope = within slices); selection criteria (*g*-index, *k* = 25), pruning (pathfinder + pruning sliced network); node types select “Author,” “Keyword,” “Institution,” “Country,” “Reference,” “Cited Author,” “Cited Journal”; selection criteria (*g*-index, *k* = 25). CiteSpace 5.8.R3 is used for visual data analysis analyzing keywords, co-cited references, and trends. A visual map created by the VOS viewer (Leiden University Science and Technology Research Centre, Leiden, The Netherlands) has nodes representing countries, institutions, authors, or keywords, which can be connected by co-authors, citations, co-citations, and co-occurrence analysis. We use the VOS viewer to perform a similar analysis of countries or regions, map their evolution, classify keywords with high co-occurrence frequencies into clusters, and create density visualization (Zhan et al. 2022).

## Results

### Publication Outputs and Trends

**Fig. 2:**
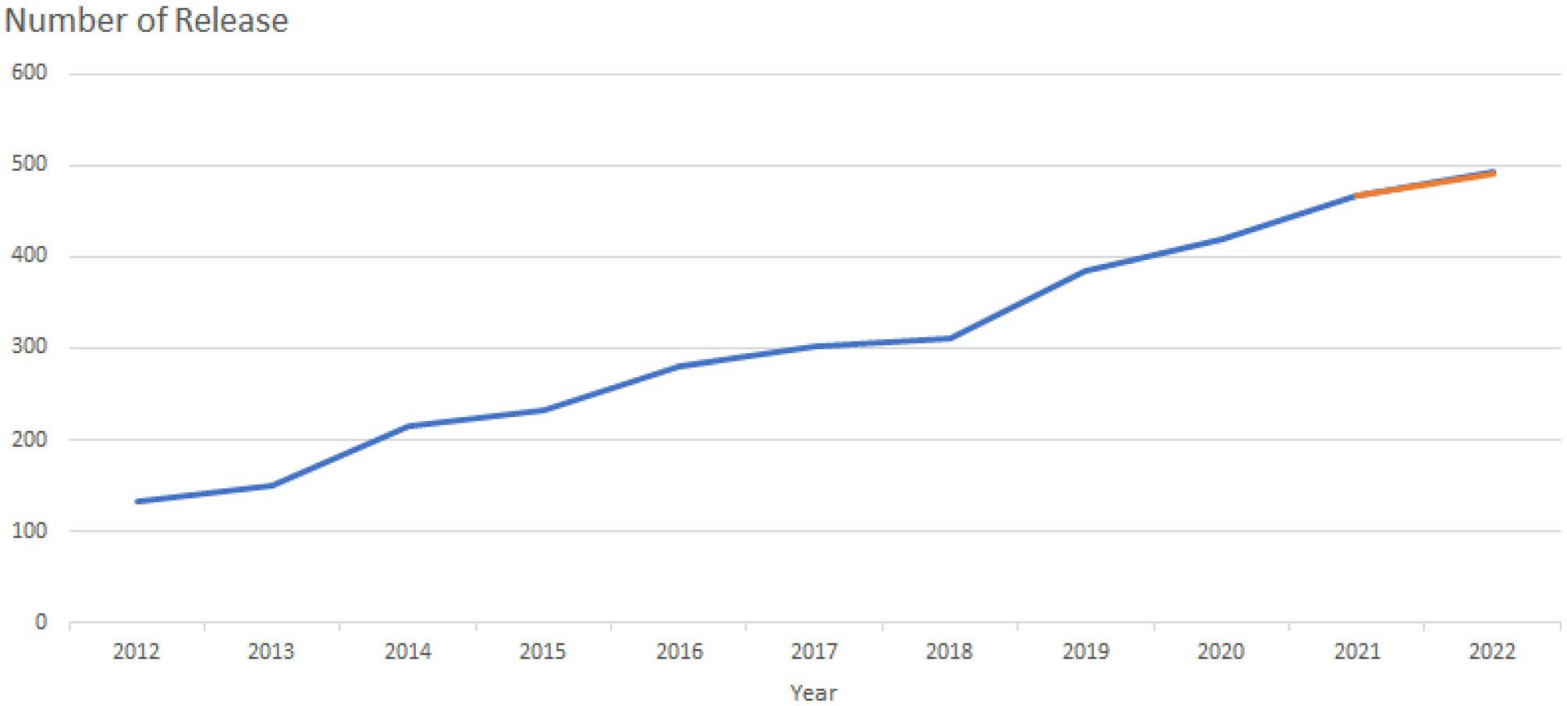
Number of annual publications on cerebral small vessel disease research from 2012 to 2022

A total of 3037 articles related to cerebral small vessel disease were retrieved. In order to investigate the research trend of cerebral small vessel disease, as shown in Fig. 2, the number of papers published each year was displayed in the form of a histogram. From 2012 to 2021, the number of related publications has increased year by year, which indicates that the research on cerebral small vessel disease is paying more and more attention. As of April 30, 2022, 143 papers have been published in 2022. The sum of the citation frequency is 53,506, and the average citation frequency of each article is 17.6.

### Fund Source 

The ten major funding sources are shown in Table 1. The National Institutes Of Health (NIH), USA, and the European Commission are the institutions with the most funding (524 and 368, respectively), followed by the National Natural Science Foundation of China (NSFC) (305), NIH National Institute of Neurological Disorders Stroke (NINDS) (256 times); funding agencies from the USA and England have provided more sponsorships in this field.

**Table 1:** Top 10 funding sources in the field of cerebral small vessel disease

Rank	Fund Source	Country/territory	Frequency
1	National Institutes of Health	USA	524
2	European Commission	European Union	368
3	National Natural Science Foundation of China	China	305
4	National Institute of Neurological Disorders Stroke Ninds	USA	256
5	UK Research Innovation	England	241
6	Medical Research Council UK, MRC	England	235
7	Ministry of Education Culture Sports Science and Technology Japan, MEXT	Japan	130
8	National Institute for Health Research	USA	117
9	National Heart Lung Blood Institute	USA	114
10	Netherlands Organization for Health Research and Development	Holland	109

### Countries and Institutions

**Fig. 3:**
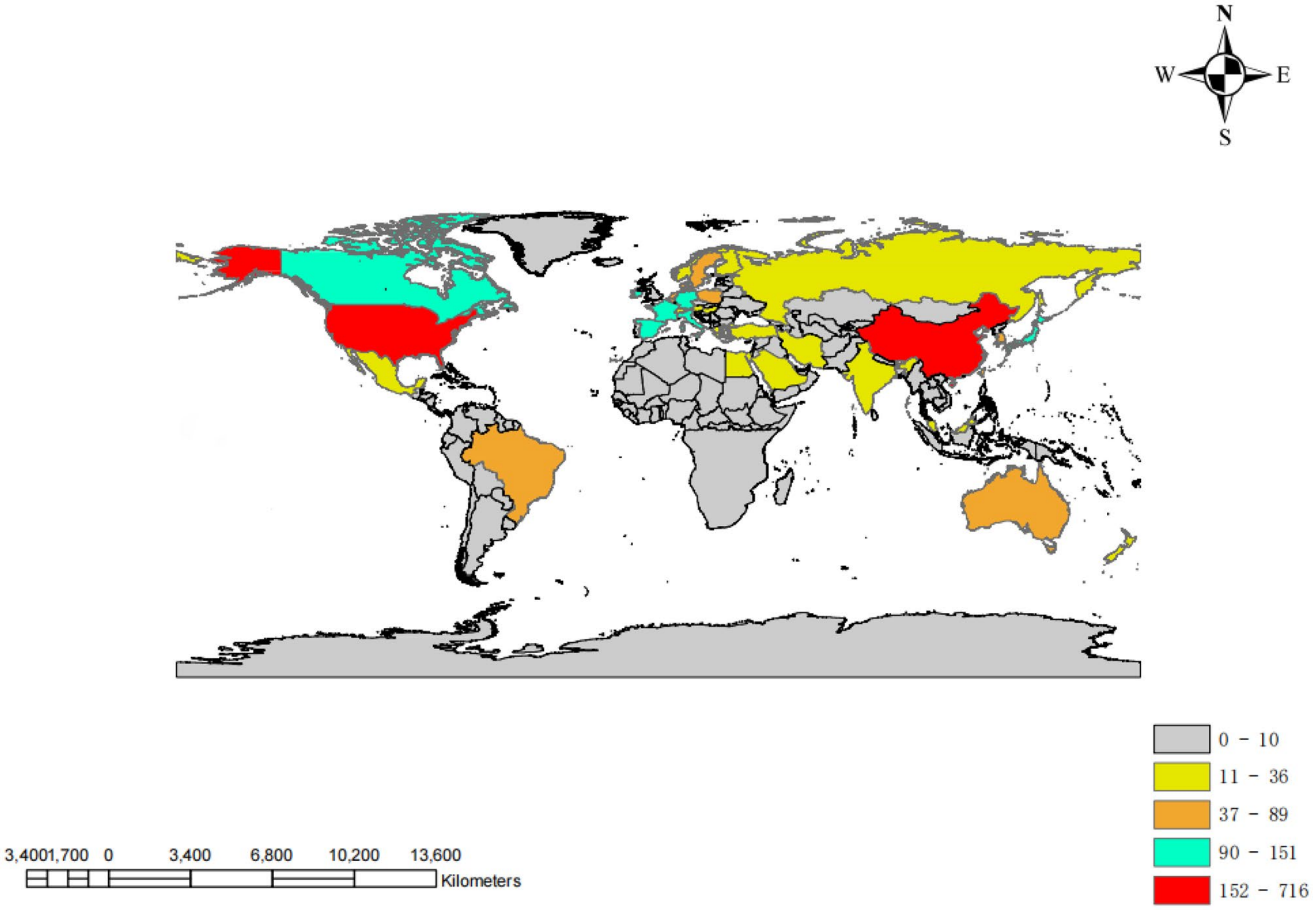
Geographical distribution map of global publications related to cerebral small vessel disease

**Fig. 4:**
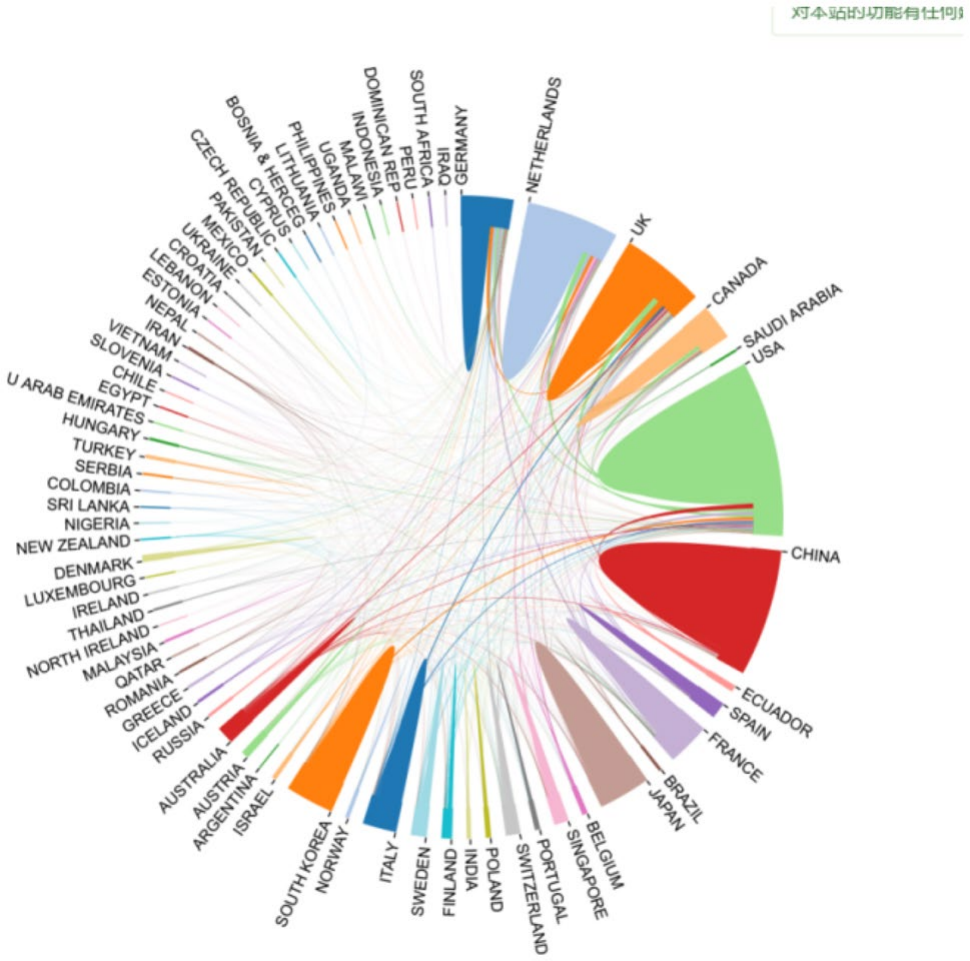
The cross-country collaborations visualization map

**Fig. 5:**
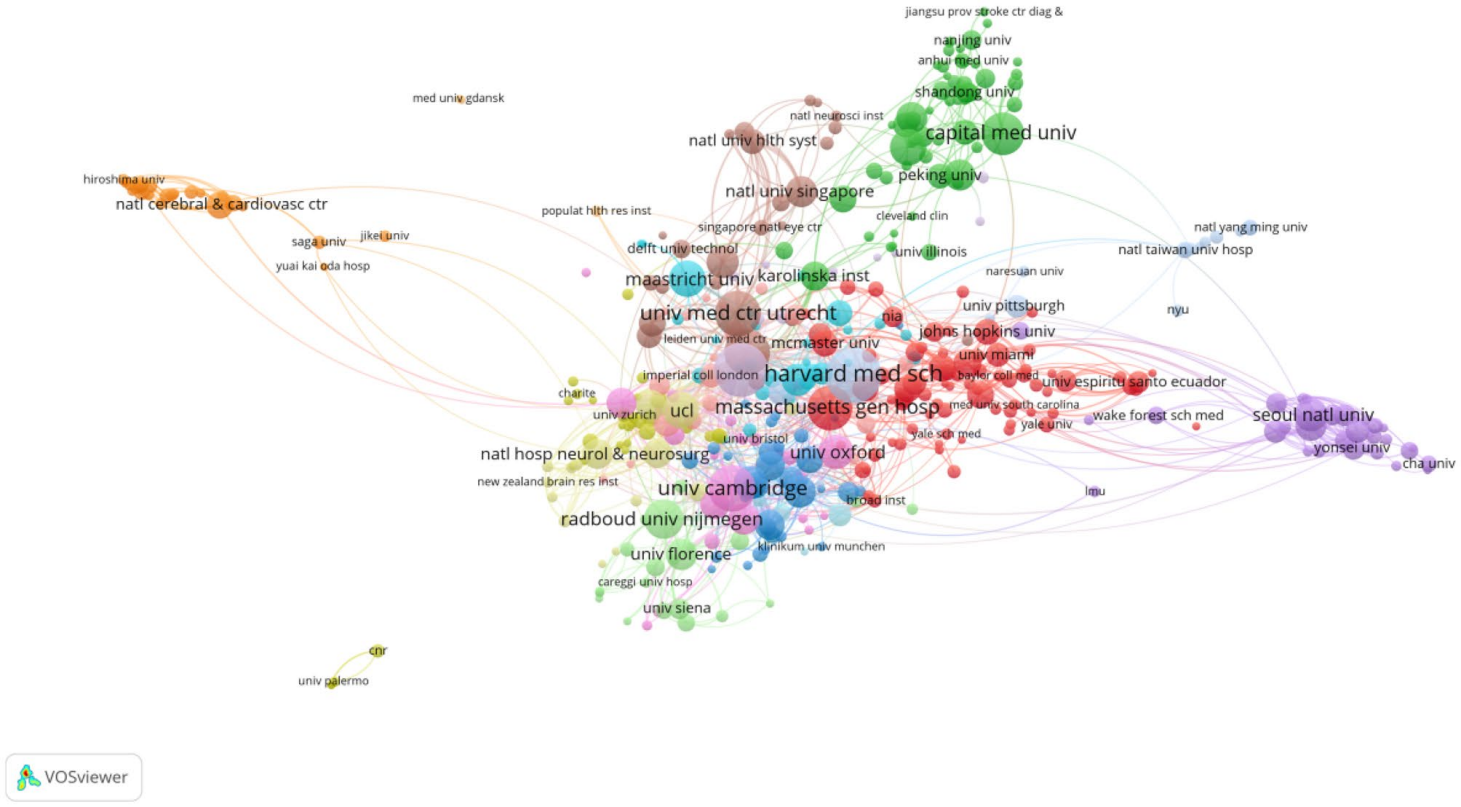
Institutional cooperation map

A map of the geographic distribution of global publications shows that articles on cerebral small vessel disease are mainly published in Europe, North America, and Asia (Fig. 3). All publications were published in 78 countries. Table 2 lists the top 10 countries and institutions for cerebral small vessel disease publications; USA is the country with the largest number of publications (800), followed by China (662 articles), and the Netherlands (424 articles), USA was cited the most (23,608 times) and reached the highest *H*-index (71). Figure 4 shows that USA attaches great importance to cooperation and has close cooperation with China, France, South Korea, and Italy. The top 10 institutions include 4 Dutch institutions, 3 UK institutions, 2 US institutions, and 1 Chinese institution. Among them, Harvard Med Sch is the scientific research institution with the largest number of published papers (137 articles), followed by the University of Edinburgh (133 articles), Univ Cambridge (104 articles), and Univ Med Ctr Utrecht (101 articles). The VOS viewer generates a network visualization map for institutional collaboration, and Fig. 5 shows that Harvard Med Sch and Univ Med Ctr Utrecht, Univ Edinburgh and Univ Cambridge, and Capital Med Univ, Fudan Univ, and Shandong Univ have a close relationship.

**Table 2:** The distribution of the top 10 countries/regions and institutions by the number of cerebral small vessel disease publications

Rank	Country/territory	Frequency	Citations	Average citation	*H*-index	Rank	Institution	Frequency	Country
1	USA	800	23,608	25.14	71	1	Harvard Med Sch	137	USA
2	China	662	8158	10.78	39	2	Univ Edinburgh	133	England
3	The Netherlands	424	12,754	27.67	56	3	Univ Cambridge	104	England
4	England	399	11,462	26.47	56	4	Univ Med Ctr Utrecht	101	The
5	Germany	290	7961	24.35	48	5	Leiden Univ	92	Netherlands
6	Japan	234	4029	14.49	33	6	Massachusetts Gen Hosp	92	USA
7	France	231	6202	24.42	42	7	Capital Med Univ	79	China
8	South Korea	195	3272	15.29	30	8	Radboud Univ Nijmegen	73	The Netherlands
9	Italy	168	3689	20.05	32	9	UCL	66	England
10	Canada	167	5050	26.58	38	10	Maastricht Univ	62	The Netherlands

### Journal Analysis

A total of 3037 articles related to cerebral small vessel disease were published in 74 journals. Table 3 lists the top 10 journals with the most articles published in cerebral small vessel disease. *Stroke* is the journal with the most articles (227 articles) (7.47%) (IF2022 = 7.19), followed by *Neurology* (4.70%) (IF2022 = 8.77), and *Journal of Stroke Cerebrovascular Diseases* (4.01%) (IF2022 = 1.787) with 143 and 122 publications, respectively. *Neurology* reached the highest *H*-index (48), with the most total citations (6301) and average citations (43.46). Among them, in the JCR partition, there are 4 in district 2, 4 in district 3, 2 in district 4, and 1 in district 1.

**Table 3:** Top 10 journals publishing research articles on cerebral small vessel disease

Rank	Journal	Frequency	Percentage	Total citations	Average citations	*H*-index	IF 2022	JCR partition
1	*Stroke*	227	7.47%	5824	25.66	42	7.19	2
2	*Neurology*	143	4.70%	6301	43.46	48	8.77	1
3	*Journal of Stroke Cerebrovascular Diseases*	122	4.01%	905	7.42	16	1.787	4
4	*Journal of Cerebral Blood Flow and Metabolism*	103	3.39%	2010	19.51	27	5.681	2
5	*Frontiers in Neurology*	99	3.25%	387	3.91	11	2.889	3
6	*Journal of Alzheimer’s Disease*	91	2.99%	1082	11.89	19	3.909	3
7	*Frontiers in Aging Neuroscience*	73	2.40%	658	9.01	15	4.362	2/3
8	*Plos One*	72	2.37%	1159	16.1	21	2.74	2
9	*International Journal*	58	1.90%	1016	17.52	18	4.882	3
	*of Stroke*							
10	*Journal of the Neurological Sciences*	55	1.81%	699	12.71	17	3.115	4

### Author Analysis

**Fig. 6:**
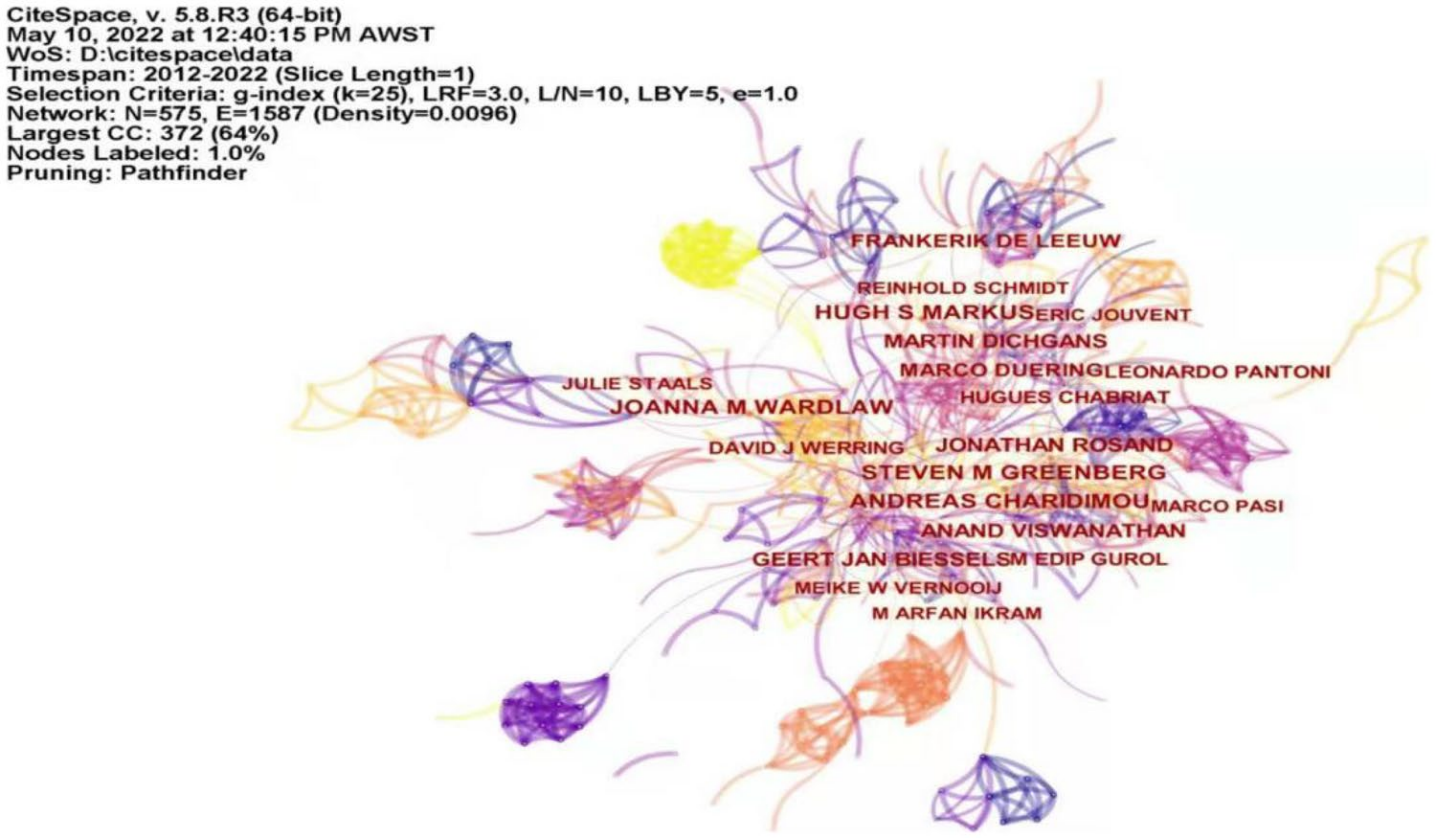
Author co-occurrence map

Among the 3037 papers related to cerebral small vessel disease research, Table 4 lists the top 10 authors with the most published papers. Andreas and Charidimou published the most papers (85 papers), followed by Joanna M. Wardlaw and Hugh S. Markus, There are 78 and 76 papers, respectively; all of them are authors who have a certain influence in the field of cerebral small vessel disease. Figure 6 shows the co-occurrence map of authors in the study of cerebral small vessel disease.

**Table 4:** Top 10 authors of cerebral small vessel disease publications

Rank	Author	Frequency
1	Andreas,Charidimou	85
2	Joanna M. Wardlaw	78
3	Hugh S. Markus	76
4	Steven M. Greenberg	74
5	Anand, Viswanathan	72
6	Jonathan, Rosand	65
7	Martin Dichgans	63
8	Geert Jan Biessels	61
9	Frankerik De Leeuw	52
10	Marco Duering	51

### Reference

**Fig. 7:**
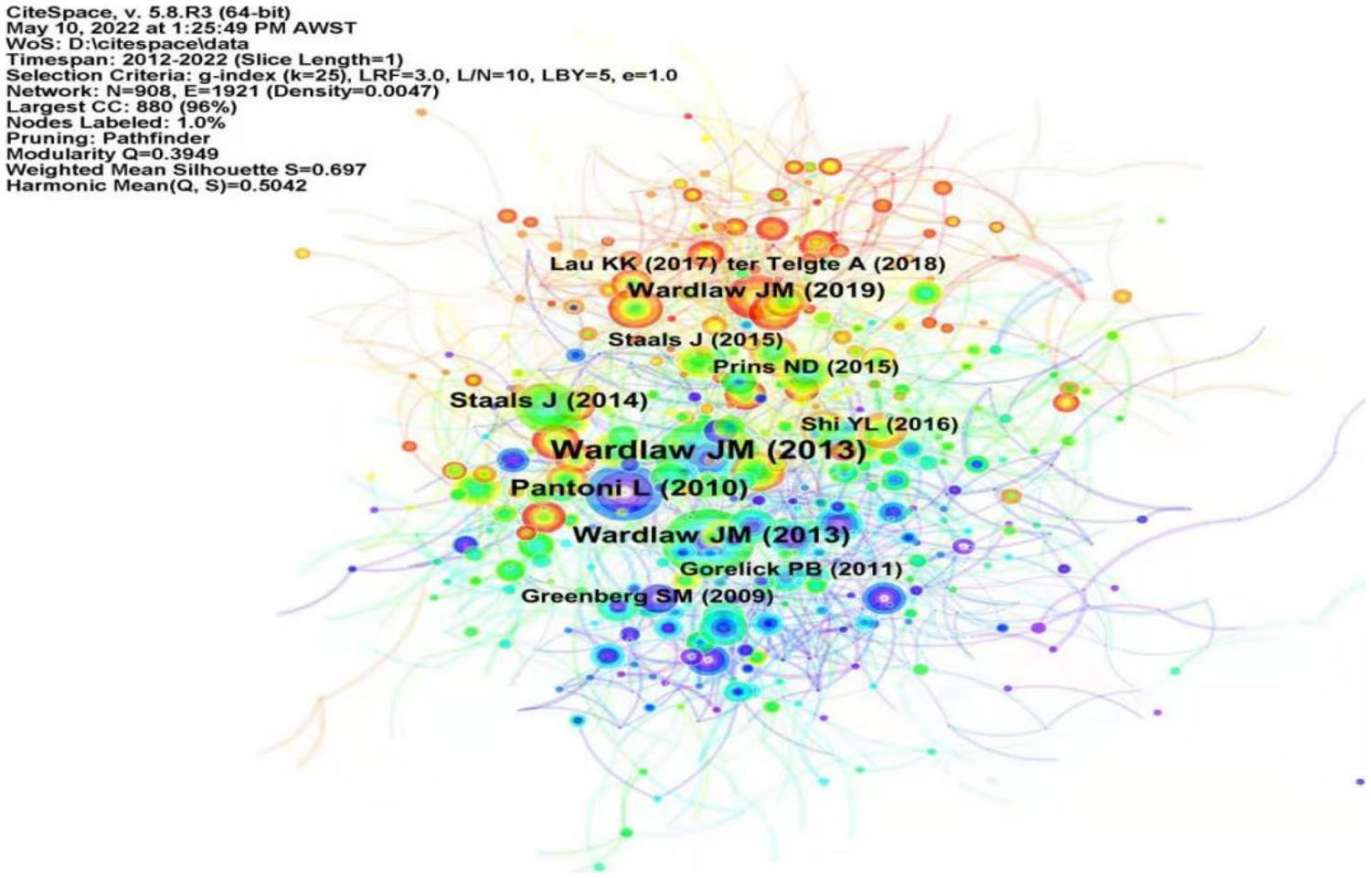
Reference co-occurrence map

From 2012 to 2022, a total of 3037 publications were visually analyzed using CiteSpace 5.8.R3, and a co-citation analysis of references was performed. After setting parameters, a visual co-citation map of the identified documents was generated. Table 5 lists the 10 most cited references during the study period, which not only laid the foundation for further research on the mechanism of cerebral small vessel disease but also provided a theoretical basis for the study of cerebral small vessel disease. The most cited paper is “Neuroimaging standards for research into small vessel disease and its contribution to aging and neurodegeneration” published by Wardlaw et al. (2013), with 438 citations. Figure 7 shows the co-occurrence map of the first author of the literature.

**Table 5:** Top 10 most cited articles in cerebral small vessel disease research

Rank	First author	Journal	Year	Citations	IDO	Centrality
1	Wardlaw, J.M	*Lancet Neurology*	2013	438	10.1016/S1474-4422(13)70124-8	0.02
2	Wardlaw, J.M	*Lancet Neurology*	2013	193	10.1016/S1474-4422(13)70060-7	0.08
3	Pantoni, L	*Lancet Neurology*	2010	172	10.1016/S1474-4422(10)70104-6	0.01
4	Wardlaw, J.M	*Lancet Neurology*	2019	129	10.1016/S1474-4422(19)30079-1	0.02
5	Staals, J	*Neurology*	2014	120	10.1212/WNL.0000000000000837	0.02
6	Prins, N.D	*Nature Reviews Neurology*	2015	72	10.1038/nrneurol.2015.10	0.03
7	Gorelick, P.B	*Stroke*	2011	68	10.1161/STR.0b013e3182299496	0.04
8	Staals, J	*Neurobiology of Aging*	2015	67	10.1016/j.neurobiolaging.2015.06.024	0.03
9	Lau, K.K	*Neurology*	2017	64	10.1212/WNL.0000000000004042	0.02
10	Shi, Y.L	*Stroke and Vascular Neurology*	2016	62	10.1136/svn-2016-000035	0.04

### Research Hotspot Analysis

#### Keyword Co-Occurrence Analysis

**Fig. 8:**
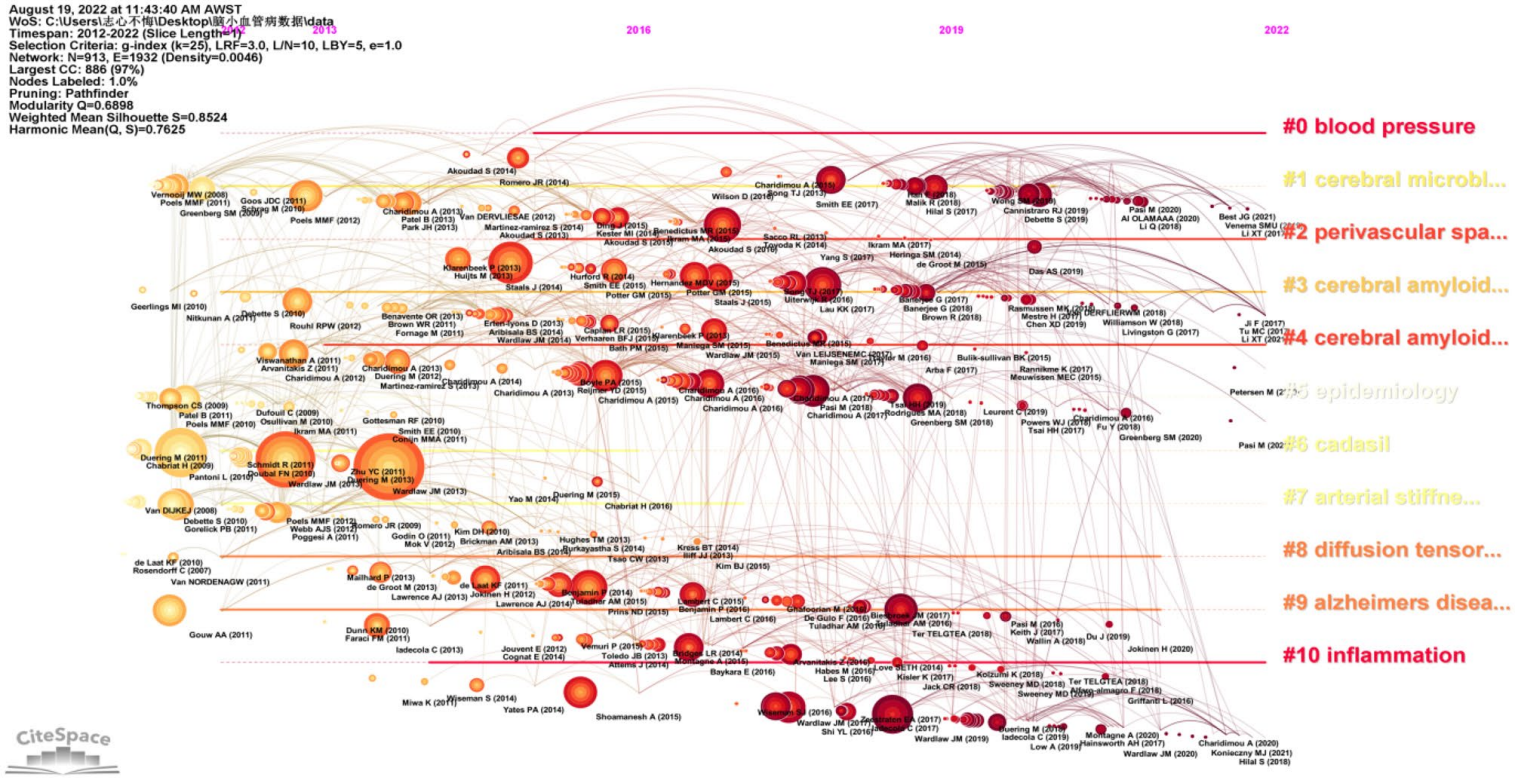
Timeline view of reference clustering. Nodes represent keywords (the bigger the circle, the more frequently it appears), and the labels of the clusters are listed on the right, where # 1 is cerebral microbleeds, # 2 is perivascular spaces, # 3 is cerebral amyloid angiopathy, # 4 is cerebral amyloid angiopathy, # 7 is arterial stiffness, # 8 is diffusion tensor imaging, and # 9 is Alzheimer’s disease

**Fig. 9:**
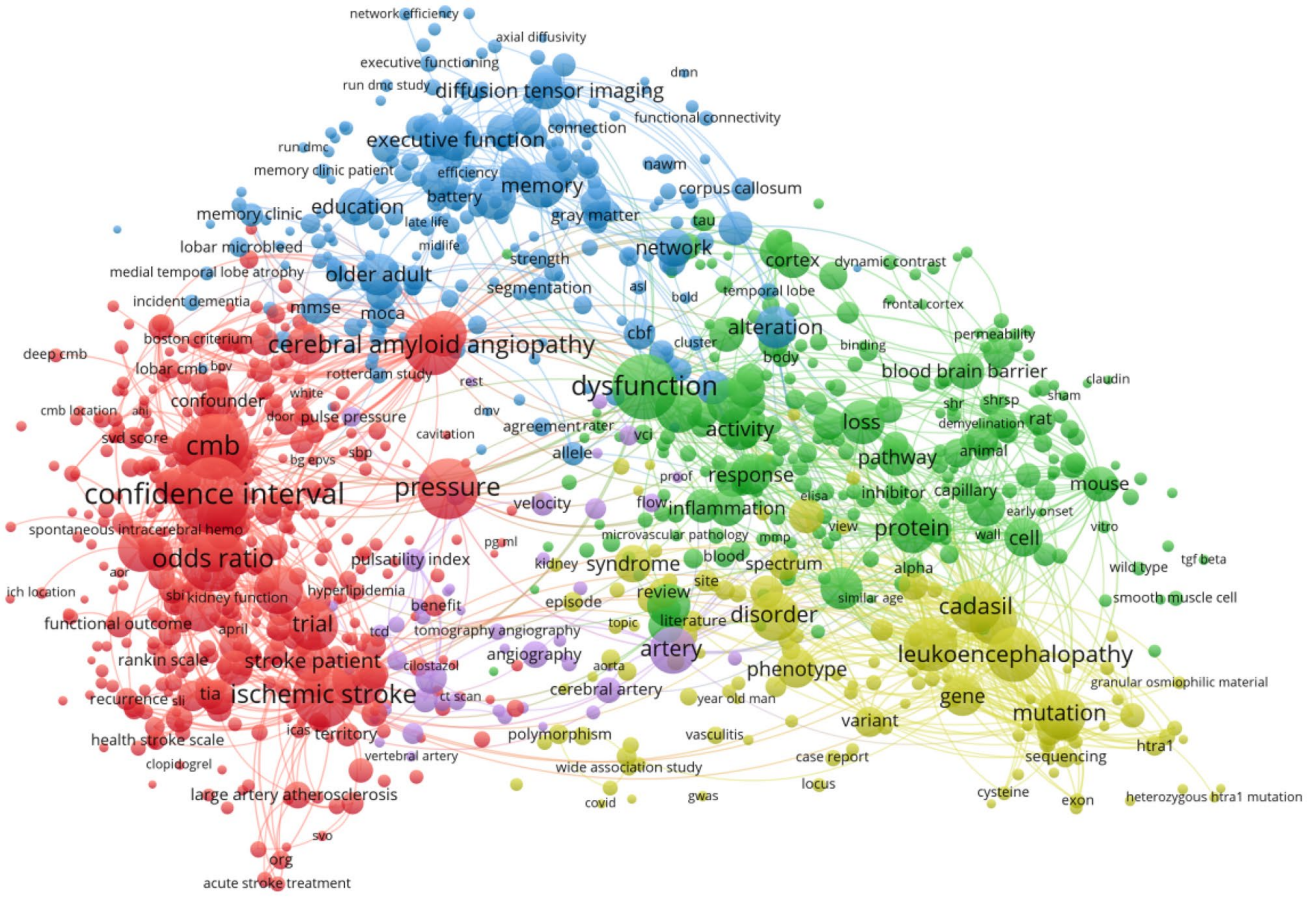
Co-occurrence map of keywords for cerebral small vessel disease research

Keywords are extracted from 3037 publications and are a very important part of the research. Keyword co-occurrence analysis provides a reasonable description of research hotspots, and emergent keywords can represent research fronts within a period of time (Xu et al. 2022) (Fig. 8). Through the network visualization analysis of keywords through VOS viewer, a keyword co-occurrence map of cerebral small vessel disease is generated, as shown in Fig. 9, and it is determined that synonyms and similar keywords will be merged in the research on cerebral small vessel disease from 2012 to 2022. After combining synonyms and similar keywords, the top 20 ranked keywords in terms of frequency can be seen (Table 6).

**Table 6:** High-frequency keywords for cerebral small vessel disease research

Rank	Keywords	Frequency	Rank	Keywords	Frequency
1	Confidence interval	390	11	Trial	182
2	Odds ratio	293	12	Disorder	182
3	Dysfunction	291	13	Intracerebral hemorrhage	180
4	Ischemic stroke	273	14	Mutation	179
5	Pressure	260	15	Cerebral autosomal dominant arteriolopathy	171
6	Cerebral amyloid angiopathy	244	16	Cerebral blood flow	165
7	Leukoencephalopathy	214	17	Artery	164
8	Infarction	212	18	Protein	162
9	Subcortical infarct	209	19	Memory	160
10	Cadasil	199	20	Vascular dementia	147

#### Keyword Cluster Analysis

**Fig. 10:**
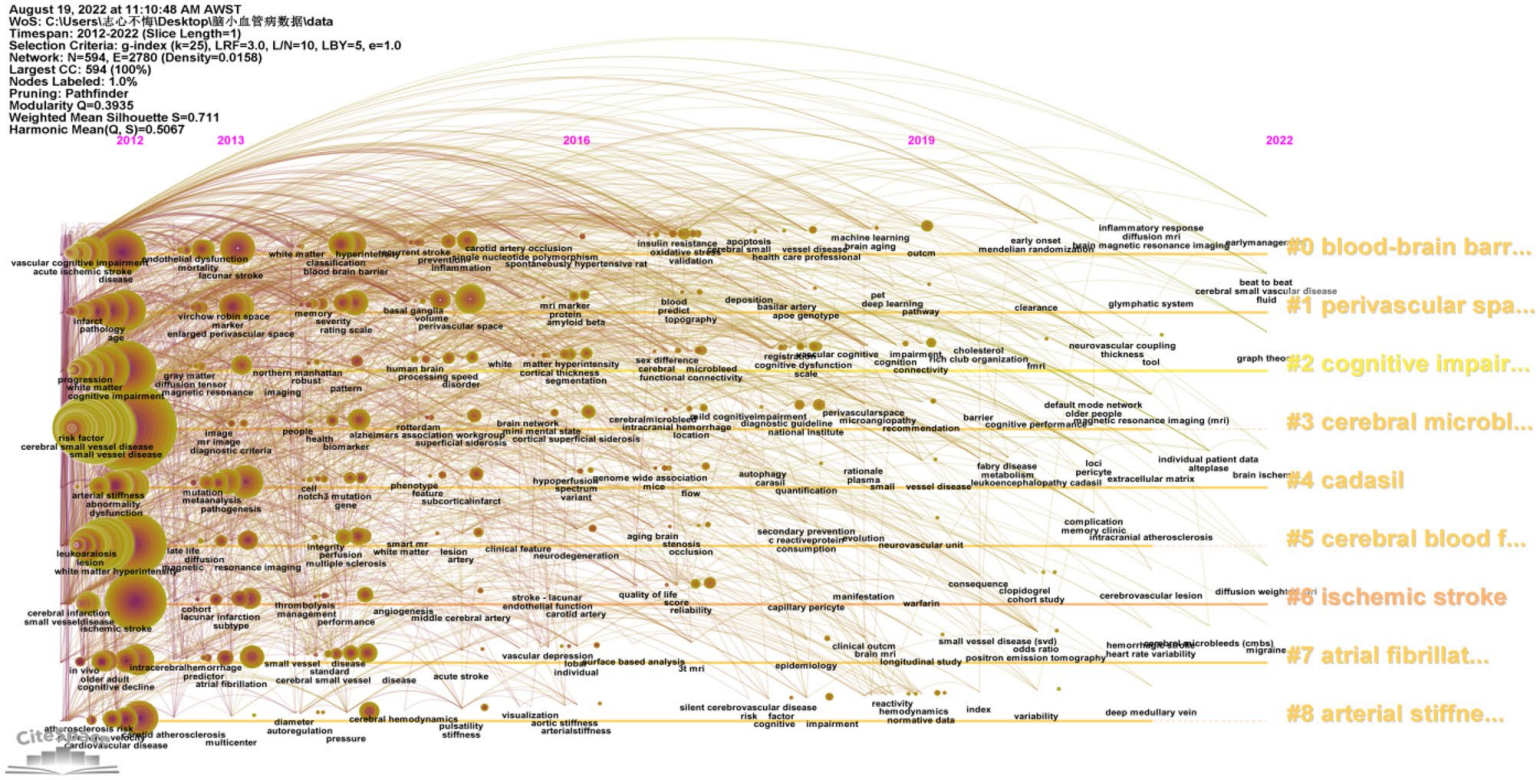
Timeline view of keywords. Nodes represent keywords (the bigger the circle, the more frequently it appears), and the labels of the clusters are listed on the right, where # 0 is a blood brain barrier, # 1 is perivascular space, # 2 is cognitive impairment, # 3 is cerebral microbleeds, # 5 is cerebral blood flow, # 7 is an atomic fiber, and # 8 is artistic stiffness

Cluster analysis is performed on the basis of keyword co-occurrence analysis, and tag clustering is performed by the likelihood ratio (LLR) algorithm (Yao et al. 2020). As shown in Fig. 10, the cluster modularity *Q* value is 0.3935, while the mean contour value *S* value is 0.711. Generally speaking, a *Q* value > 0.3 represents a significant clustering structure; if the average cluster contour value *S* > 0.5, the clustering is generally considered to be reasonable. If the *S* value > 0.7, the clustering result is considered convincing. A total of 9 clusters were formed by keywords in this study, which to a certain extent demonstrated the knowledge structure and dynamic change process in the field of cerebral small vessel disease.

Cluster analysis was conducted according to the similarity of keywords. The results showed that the current research on cerebral small vessel disease mainly focuses on three aspects: ① #0 and #7 are disease research fields, mainly related to the sequelae caused by cerebral small vessel disease, including keywords such as stroke, dementia, cognitive impairment, etc.; ② #1, #3, and #4 are pathological changes of cerebral small vessel disease, mainly including small arteriosclerosis, sporadic and hereditary cerebral amyloid angiopathy, and other hereditary small vessel disease; ③ #2, #5, #6, #8, and #9 are the influence changes caused by cerebral small vessel disease (Harshfield et al. 2022), including lacunar cerebral infarction, white matter lesions, cerebral microbleeds, enlarged perivascular space, and brain atrophy and recent small subcortical infarct (see Table 7).

**Table 7:** Keyword cluster analysis

Cluster ID	Size	Silhouette	Mean (year)	Top terms (LSI)
#0	70	0.583	2015	Blood pressure; major depressive disorder; cognitive decline
#1	68	0.637	2015	cerebral microbleeds; amyloid-beta protein precursor; blood–brain barrier
#2	63	0.713	2015	perivascular spaces; white matter hyperintensities; diffusion tensor imaging; deep gray matter; brain atrophy
#3	62	0.684	2014	cerebral amyloid angiopathy; arterial stiffness
#4	62	0.702	2015	cerebral amyloid angiopathy; pulse wave velocity; pulsatility; population-based study; small vessel disease score; risk factors; pressure
#5	61	0.648	2015	epidemiology; white matter; perivascular space; atherosclerosis
#6	60	0.823	2013	cadasil; normal-appearing white matter; white matter hyperintensities; enlarged perivascular spaces
#7	59	0.642	2014	arterial stiffness; ischemic stroke; cognitive screening; transient ischemic attack; acute ischemic stroke
#8	48	0.75	2016	cerebral small vessel disease; subcortical infarct; leukoencephalopathy
#9	32	0.826	2016	Alzheimer’s disease; perivascular space; glymphatic system; global cerebral atrophy

#### Keyword Co-occurrence Analysis

**Fig. 11:**
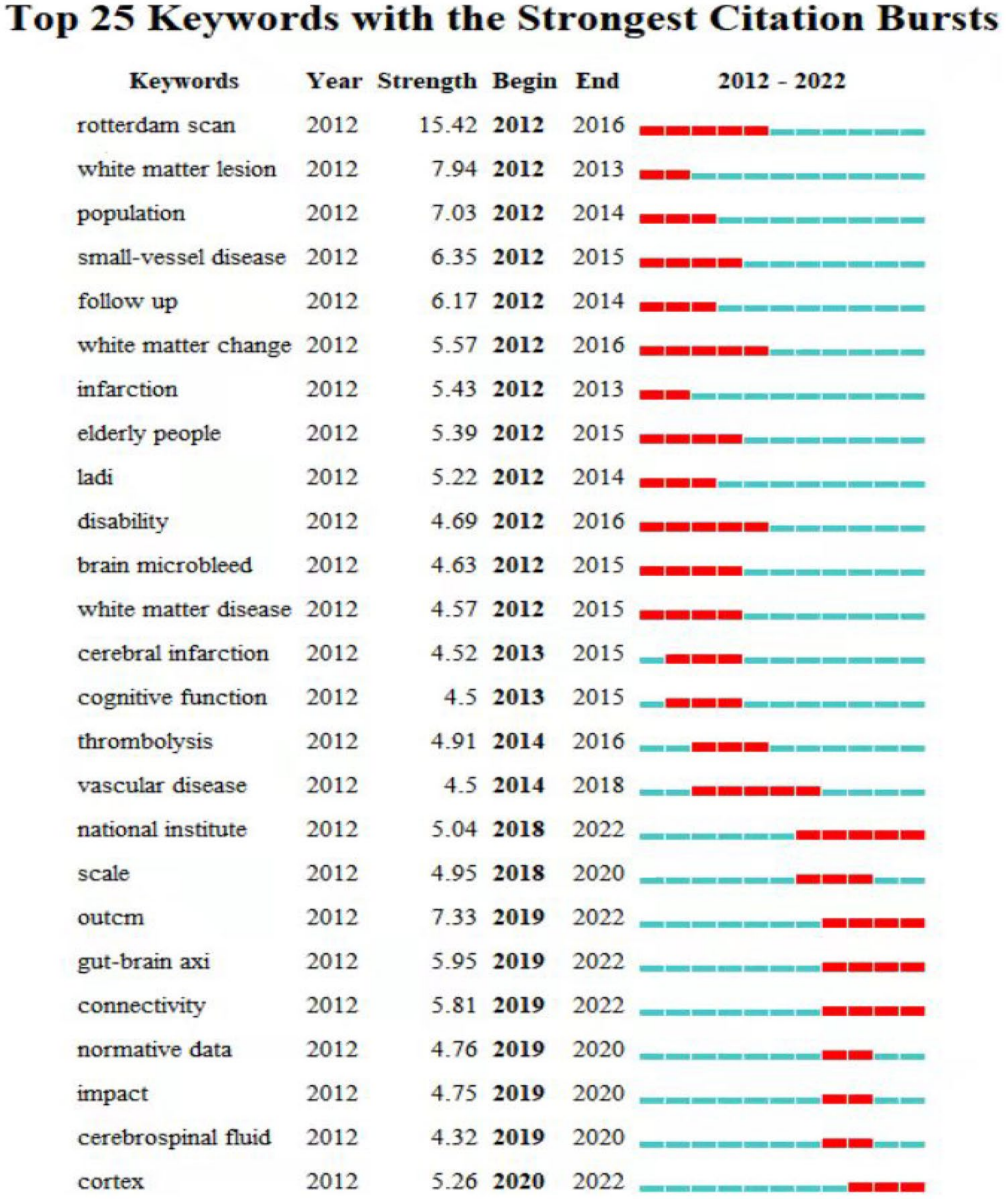
Keyword emergence analysis in the literature on cerebral small vessel disease

According to the detection of emerging words with high frequency and fast growth rate within a period of time, keywords with high-burst intensity are an important indicator reflecting research hotspots, frontiers, and latest trends (Fig. 11). A total of 25 emergent words were detected, and the Rotterdam scan had the highest burst intensity (intensity = 15.42), followed by white matter lesion (intensity = 7.94), and outcm (intensity = 7.33). It is worth noting that “national institute” (2018–2022, 5.04), “outcm” (201–-2022, 7.33), “gut-brain axi” (2019–2022, 5.95), “connectivity” (2019–2022, 5.81), “cortex” (the citation burst of keywords such as 2020–2022, 5.26) continued until 2022, indicating that these research directions are likely to become new research hotspots in the future.

## Discussion

### General Information

We performed a bibliometric analysis of 3037 articles related to cerebral small vessel disease. As shown in Fig. 2, the number of global publications on cerebral small vessel disease has shown a steady upward trend. Currently, 143 articles have been published in 2022, although the data for 2022 is incomplete, and it is expected that the production of articles in 2022 will increase, which indicates that research related to cerebral small vessel disease is increasingly attracting the attention of scholars.

Bibliometric analysis takes the global literature pattern and literature characteristics as research objects, including countries, institutions, authors, and journals over a period of time (Liu et al. 2019). In terms of country/regional analysis, research centers in this field are concentrated in Europe, North America, and Asia. The USA is the most productive country, with a much higher number of publications than other countries/regions, which is closely linked to funding sponsorship by funding agencies. In terms of research institutions, strengthening the cooperation between different institutions or teams is extremely important for future basic or clinical trials of cerebral small vessel disease. Among the published journals, *Stroke*, *Neurology*, and *Journal of Stroke Cerebrovascular Diseases* are the top 3 journals with the most publications. Andreas, Charidimou, Joanna M. Wardlaw, and Hugh S. Markus are among the top 3 authors in the field of cerebral small vessel disease by volume.

### Bibliography Analysis

Co-citation analysis is generally used as a method to evaluate the academic influence of journals or scholars (Di Donato et al. 2017), who are all authors with certain academic influence (Dong et al. 2022). Wardlaw et al. published the most influential papers with the most citations (438 times). Through further research, it was found that Wardlaw et al. (Dong et al. 2022) published several articles mainly on the mechanism, clinical significance, and aging and neurodegeneration of cerebral small vessel disease. Lau et al. (2017) found that a higher total CSVD score was associated with an increased risk of recurrent ischemic stroke. In therapy, Wardlaw et al. (2019), (Lau et al. 2017) found emerging targets for new therapies including brain barrier integrity, vascular reactivity, vascular compliance, perivascular inflammation, or myelin repair. Shi and Wardlaw (2016) believed that new research should consider drugs targeting the endothelium and blood–brain barrier to prevent and treat CSVD. At the same time, changing traditional risk factors and healthy lifestyles are also an important prevention and treatment method.

### Hotspots and Frontiers

In terms of different forms of coexisting disease of CSVD, as Alzheimer’s disease and cerebral small vessel disease (CSVD) often coexist (Chojdak-Lukasiewicz et al. 2021), the interaction between the two is unclear (Kim et al. 2020). Pathologically, wall damage can lead to external expansion of microaneurysms due to fibrosis and proximal lumen stenosis or obstruction (Chou et al. 2021). Ultimately, impaired autoregulation of the involved small vessels results in decreased cerebral blood flow and chronic cerebral hypoperfusion (Litak et al. 2020), and occlusion of the arterial lumen leads to acute ischemia, leading to lacunar infarction (Tang and Liu 2021; Kim and Lee 2015). Severe stenosis and hypoperfusion involving multiple arterioles, mainly deep white matter, leading to incomplete ischemia, are seen on neuroimaging as “white matter hyperintensity” (WMH) (Wang et al. 2021; Biesbroek et al. 2017). The two pathophysiological pathways above may often overlap, so lacunar and white matter lesions often coexist. CSVD studies the coexistence of different forms, and this field has received less attention and should be strengthened.

### Research on Cerebral Small-Vessel Disease and White Matter Disease

The main symptoms of CSVD patients include lower extremity motor dysfunction characterized by gait disturbance and cognitive dysfunction characterized by impaired executive function (Wang et al. 2021; Biesbroek et al. 2017). White matter lesions (WMLs) are more common in the elderly. The symptoms of WMLs are subtle, such as cognitive impairment, dementia, and depression (Manyangu et al. 2019; Putilina 2019). They are usually symmetrically distributed in the white matter including the pons and brain stem on imaging, and also in the deep gray matter (Wang et al. 2020; Hashimoto et al. 2016). White matter hyperintensity (WMH) is the most important predictor of gait dysfunction, and more severe WMH-related defects are located in the internal capsule, hemi-egg center, periventricular frontal lobe, etc. (Kang et al. 2022; Wang et al. 2022). The number of lacunar infarcts is a predictor of executive dysfunction, presumably due to damage to the frontal-subcortical circuit, reduced connectivity and metabolism within the prefrontal cortex, and ultimately executive dysfunction (de Laat et al. 2011). However, the pathogenesis of white matter disease (WML) and lacunar infarction has not yet been fully explained. The role of cortical thickness remains unknown. There is a lack of attention in this area, and cortical studies in cerebral small vessel disease should be strengthened (de Leeuw et al. 2002).

### The Effect of Brain-Gut Axis on Cerebral Small-Vessel Disease

Gut-brain axis is a bidirectional pathway between the gastrointestinal system and the central nervous system (He et al. 2018), involving nerves, endocrinology, immunity, etc. The gut microbiota has a symbiotic relationship with enterocytes and plays an important role in digestion (Markus 2021). The gut microbiota maintains bidirectional interactions with major parts of the central nervous system through direct and indirect pathways (Tonomura and Gyanwali 2021; Nelson et al. 2021). Microbial components may enhance systemic inflammation and amyloid fibril formation, ultimately leading to amyloid deposition and brain microvascular systemic inflammation caused by gut microbiome or microbiome metabolites may affect brain parenchyma (Camara-Lemarroy et al. 2018). Studies have shown that the gut microbiome is associated with characteristics suggestive of the presence of CSVD, and that the gut microbiome or its metabolites may influence the presence of CSVD through the microbiome gut-brain axis (Niemarkt et al. 2019). The influence of the brain-gut axis on cerebral small vessel disease is a new direction. At present, there are few studies in this field, and more attention should be paid to this field.

### Study on Drug Intervention of Intestinal Flora in the Treatment of Cerebral Small-Vessel Disease

We found a role for chronic systemic inflammation in the pathophysiology of CSVD. Systemic and vascular inflammatory states have been found to be associated with the development and prognosis of CSVD (Bouasquevisque et al. 2019). The peripheral immune system, including innate and adaptive immune cells, plays a crucial role in the pathophysiology of CSVD (Cardona and Escrig 2018). Neutrophils infiltrate CSVD lesions across the blood-brain barrier (BBB), and their sustained release of the BBB to disrupt matrix metalloproteinase 9 (MMP9) has been identified (Tonomura and Gyanwali 2021). The researchers found that circulating monocytes in patients with CSVD had increased cytokine production capacity (Yang et al. 2022). The release of central nervous system (CNS) antigens into the peripheral circulation leads to the development and activation of CNS antigen-specific lymphocytes. Multiple inflammatory mediators derived from activated immune cells contribute to the development of CSVD (Zanon et al. 2021; Zhou et al. 2021). Systemic and vascular inflammatory factors synergistically drive the progression of small vessel disease (Tonomura and Gyanwali 2021). Addressing the immune response of injured brain endothelium and the BBB may be a promising therapeutic strategy (Cai et al. 2021). Ongoing interactions between the gut microbiota and the host immune system have important implications for the induction, functional modulation, or suppression of local and systemic immune responses (Jian et al. 2020). The field of drug intervention of intestinal flora in the treatment of cerebral small vessel disease is almost blank, and it is also one of the main research trends in the future. Research in this field should be strengthened. The treatment of small vessel disease has more options and may have breakthrough effects.

## Conclusion

We analyzed the research progress, hotspots, and frontiers in this field; studied cerebral small vessel disease through bibliometric analysis; and revealed the future research prospects. Currently, research on cerebral small vessel disease is in a rapid development stage, and since 2012, publications related to cerebral small vessel disease have steadily increased. At the same time, we identified leading countries, institutions, and leading scholars in the field and analyzed journals and representative literature. Cortex and gut-brain axis are the latest research hotspots through keyword co-occurrence analysis and burst graph emergence detection. It is worth noting that the mechanism of cerebral small vessel disease is still unclear and needs further study. While this study searched the largest database, it may have missed some studies beyond the scope of the database. Future reviews may search multiple databases. Despite its limitations, this novel study is the first to synthesize nearly 10 years of cerebral small vessel disease research and will serve as an important addition to the existing knowledge base. In conclusion, bibliometric analysis provides objective insights into the research of cerebral small vessel disease, full of opportunities and challenges.
